# The Institutional Worker

**Published:** 1911-11-11

**Authors:** 


THE
### INSTITUTIONAL WORKER
Being a Special Supplement to "The Hospital"

### Editor's Notices.

**Contributions:**

Contributions should be written, or preferably typed, on one side of the paper only, and all articles sent in are accepted upon the distinct understanding that they are forwarded to The Hospital only.

The Editor cannot undertake to return MSS. not used, but when a stamped directed envelope is enclosed the MSS. may be returned if a special request is made.

Accepted articles and paragraphs of news will be paid for after publication at the scale rate.

**Address:**

To prevent delay all contributions and letters on editorial business must be addressed exclusively to the Editor, "The Hospital," 29 Southampton Street, Strand, London, W.C. It is important that this regulation shall be strictly observed.

**Correspondence:**

Correspondence on all subjects is invited. The name and address of correspondents must be given as a guarantee of good faith, but not necessarily for publication.

**Special Articles:**

Special articles are invited, and questions, inquiries, and paragraphs upon all matters relating to the work, administration, and management of general, special, mental, fever, cottage and convalescent hospitals, sanatoria, homes, institutions, societies and organisations for the treatment or care of the sick, injured and dependents of all classes. Every matter affecting the interests and welfare of the staff of all grades working in these institutions will receive special consideration.

**Administrative Medicine, Research and Items of News:**

Special terms will be paid for approved articles on subjects in this wide field by experts who have made some section of it their special study and interest. We wish to give prominence to every movement tending to advance accuracy of diagnosis, efficiency in treatment, and the development of modern methods to eradicate disease and promote the welfare of the suffering.

**A Bureau of Information:**

We invite inquiries and applications for information and help in providing for that numerous class of sufferers who require special care or house-room which they cannot obtain in their own homes or secure for themselves. **We cannot, however, prescribe or recommend practitioners.**

**Books for Review:**

Publishers are particularly requested to send advance proofs of any new books of importance whenever possible, as the Editor has made arrangements to publish immediate reviews on a new plan.

**Photographs, Plans, Blocks and Illustrations:**

It is requested that wherever possible MSS. may be accompanied by illustrations in any of the above forms. The name of the sender and of the article to which it belongs should be written on the back of each photograph, drawing, or block for purposes of identification.

**Local Papers:**

Newspapers containing reports or news paragraphs **should be marked** and addressed to the Sub-Editor.

**The Coupon System:**

A coupon will be found at the bottom of the third inside page of the cover each week. This coupon must be attached to every question or inquiry to which an answer is desired in The Hospital.

### Manager's Notices.

Letters relating to the publication, sale, and advertising departments of The Hospital must be addressed to the Manager, "The Hospital," The Hospital Building, 28 and 29 Southampton Street, Strand, London, W.C.

**Circulation and Sale:**

The Hospital is on sale at all newsagents and bookstalls every Friday, price one penny. The rates of subscriptions, post free from The Hospital office, have been reduced and are now as follows :—

| | United Kingdom s. d. | The Colonies and Abroad s. d. |
|---|---|---|
| For one year ... ... ... | 6 6 | 8 8 |
| ,, six months ... ... | 4 0 | 5 0 |
| ,, three months ... ... | 2 0 | 2 6 |

**Subscriptions,** which may begin at any time, are payable in advance. Cheques and Post-Office orders, crossed London County and Westminster Bank, Covent Garden branch, should be made payable to the Manager of The Hospital and addressed to him at The Hospital Building, 28 and 29 Southampton Street, Strand, London, W.C.

**Advertisements:**

All advertisements for The Hospital must reach the Manager not later than Wednesday morning in each week. For scale of charges see pages v and 2.

**Classified Advertisements:**

All small advertisements will be classified, for this section of the paper is widely read and of special interest. We wish to encourage those wanting appointments who are attracted to institutional work, and therefore offer them the reduced rate of *twenty-one words and under for* 1s., and *for every ten and part of ten words beyond*, 6d.

### Points for Institution Authorities.

The economical management of an Institution depends very greatly upon purchasing supplies in the cheapest market.

It is often impossible locally to secure the highest quality at the lowest prices.

In consequence, a section will be reserved in this part of the paper for announcements of Institutions inviting Tenders for Contracts.

This will enable Authorities of Institutions to secure tenders for supplies from all over the country, and ensure purchases at the lowest possible prices consistent with quality.

The charge for Tenders is 6s. per inch, and for this small outlay many pounds may be saved on supplies of all descriptions.

**Special Rates** will be quoted for those institutions which agree to send all their vacancy, contract, and public notices to The Hospital each year, so as to make it a file of such announcements for ready reference.

### Points for Trade Advertisers.

The Hospital is the organ of Institutions, whether of a Voluntary Character or those under Local Government, Poor Law and Municipal Authorities.

It is read by all Executive Officials and those responsible for the Construction, Management, and Equipment of Public Establishments.

It is also the Journal of the worker engaged in the Medical, Health and Preventive services.

There is no medium more appropriate and effective in the promotion of business in these directions than The Hospital.

It is the Authoritative Journal of Administrative Medicine and of Institutional Life, and is relied upon by all connected with or interested in the Prevention of Disease, the alleviation of the sick, and the general health and well being of the community.

### APPOINTMENTS VACANT.

### Poor-Law Vacancies.

### Appointments Vacant.

Assistant Labour Master (£28), Woolwich Union. Mr. T. Cutter, C. to G., Woolwich. Nov. 13.

Assistant Nurse (£25), Louth Union. Mr. F. C. Chard, C. to G., Louth. Nov. 18.

Assistant Nurse (£25), Guildford Union. W. S. V. Cullerne, C. to G., Commercial Road, Guildford. Nov. 24.

Assistant Nurse (£25), Wortley Union. J. Norton, C. to G., Union Offices, Grenoside, Sheffield. Nov. 15.

Assistant Nurse (£25), Belper Union. J. Pym, C. to G., Belper. Nov.17.

Assistant Nurse (£28), Eastry Union. F. S. Cloke, C. to G., Sandwich. Nov. 20.

Assistant Nurse (£20), Bideford Union. M. J. Durant, C. to G., Bideford. Nov. 13.

Assistant Nursery Attendant (£18), Lewisham Union. Mr. H. C. Mott, C. to G., 394 High Street, Lewisham. Nov. 11.

Case Paper Clerk (32s. weekly), Woolwich Union. Mr. T. Cutter, C. to G., Woolwich. Nov. 14.

Charge Attendant on Male Lunatics (£50), Kingston Union. Mr. C. W. Dash, C. to G., Kingston-on-Thames.

Charge Nurse (£30), Pontypridd Union. Mr. W. Spickett, C. to G., Pontypridd. Nov. 14.

Charge Nurse (£32), Huddersfield Union. Mr. E. A. Rigby, C. to G., Huddersfield. Nov. 15.

Charge Nurse (£30), Chesterfield Union. R. F. Hartwright, C. to G., Union Offices, Chesterfield. Nov. 16.

Charge Nurse (£32), Middlesbrough Union. J. J. Dales, C. to G., Poor Law Offices, Middlesbrough. Nov. 22.

Charge Nurse (£30), Mutford and Lothingland Union. F. Peskett, 148 London Road, Lowestoft. Nov. 17.

Charge Nurse (£24), City of London Union. Matron of the Infirmary, 42 Clifden Road, Median Road, N.E.

Charge Nurse (£30), Plymouth Guardians. W. H. Davy, C. to G., Greenbank Road, Plymouth. Nov. 17.

Female Attendant (£18), Bakewell Union. Mr. A. Hawes, C. to G., Bakewell. Nov. 11.

Female Dispenser, Etc. (£75 non-resident), Croydon Union. Mr. H. List, C. to G., Mayday Road, Thornton Heath. Nov. 13.

Foster-Mother (£20), Stoke-on-Trent Union. Mr. T. Rowley, C. to G., Stoke-on-Trent. Nov. 16.

Foster-Mother (£22), Wallingford Union. Mr. G. F Slade, C. to G., Wallingford. Nov. 16.

Head Female Attendant (£40), Birmingham Guardians. Mr. C. Fletcher, C. to G., Birmingham. Nov. 13.

Head Male Attendant (£40), Sheffield Union. Mr. A. E. Booker, C. to G., Sheffield. Nov. 11.

Head Nurse (£32), Southwark Union. Mr. S. Wood, C. to G., Ufford Street, Blackfriars, S.E. Nov. 13.

Junior Clerk (£50), Brentford Union. Mr. W. Stephens, C. to G., Isleworth, W. Nov. 14.

Labour Master (£35), Woolwich Union. Mr. T. Cutter, C. to G., Woolwich. Nov. 13.

Male Industrial Trainer (Shoemaker or Tailor), Barnsley Union. Mr. C. J. Tyas, C. to G., Barnsley. Nov. 13.

Master and Matron (£100 and £50), Barnsley Union Mr. C. J. Tyas, C. to G., Barnsley. Nov. 13.

Master and Matron (£70 and £50), Fylde Union. Mr. F. H. Brown, C. to G., Wesham, Kirkham. Nov. 13.

Master and Matron (£70 and £40), Bridgwater Union. Mr. T. M. Reed, C. to G., Bridgwater. Nov. 17.

Master and Matron (£60 and £40), Lancaster Union. Mr. W. D. Ball, C. to G., Lancaster. Nov. 13.

Massage Sister (£36), Birmingham Infirmary, Dudley Road. Apply Matron. Nov. 13.

Matron and Caretaker (husband and wife), Stanhope and Weardale Joint Hospital Committee. F. H. Thompson, Clerk, Stanhope, Co. Durham. Nov. 15.

Nurse (£25), Strand Union. Mr. A. H. Maddocks, C. to G., 15 Henrietta Street, Covent Garden, W.C. Nov. 15

Nurse (£35), Sleaford Union. Mr. E. Clements, C. to G., Sleaford.

Nurse (£27), Castle Ward Union. C. S. Shortt, C. to G., 18 Bigg Market, Newcastle-on-Tyne. Nov. 13.

Probationer (£12), Hunslet Union. Mr. F. W. Mee, C. to G., Hunslet, Leeds. Nov. 15.

PROBATIONERS (£10), Wakefield Guardians. H. Beaumont, C. to G., 47 Kirkgate, Wakefield. Nov. 25.
PROBATIONERS (£8), Chesterfield Union. R. F. Hartwright, C. to G., Union Offices, Chesterfield. Nov. 16.
PROBATIONER (£12), Mitford and Launditch Union. Matron of the Workhouse, Gressenhall, East Dereham.
PROBATIONER NURSE (£10), Burton-on-Trent Union. C. F. Chamberlin, C. to G., Union Offices, Burton-on-Trent. Nov. 20.
PROBATIONER NURSES (£10), Wisbech Union. T. H. Stockdale, C. to G., 2 Ely Place, Wisbech. Nov. 13.
PROBATIONER NURSES, Kingston-upon-Hull Guardians. R. H. Winter, C. to G., St. Mary's Chambers, Hull. Nov. 18.
RELIEVING OFFICER AND COLLECTOR (£120 and Commission), Worcester Union. Mr. J. G. Shield, C. to G., Worcester. Nov. 16.
SECOND ASSISTANT MASTER'S CLERK (£25), Willesden Union. Mr. J. Hutton Haylor, C. to G., 357 High Road, Brondesbury, N.W. Nov. 14.
SISTER (£30), Halifax Union. Matron, St. Luke's Hospital, Halifax.
SISTER (£35), Wandsworth Union. Mr. F. W. Piper, C. to G., St. John's Hill, Wandsworth, S.W.
STAFF NURSES (£31), West Ham Union. Mr. T. Smith, C. to G., Leytonstone, N.E. Nov. 23.
STAFF NURSE (£30), Hackney Union, Matron, Union Workhouse, Sidney Road, Homerton, N.E.
STAFF NURSE (£24), Hackney Union. Matron of the Workhouse, Sidney Road, Homerton, N.E.
TEMPORARY CLERK (£2 weekly), Brentford Union. Mr. W. Stephens, C. to G., Isleworth, W.
THIRD ASSISTANT BOOKKEEPER (£30), South Manchester Guardians. Mr. D. S. Bloomfield, C. to G., All Saints', Manchester.
WARD SISTER (£30), Hammersmith Guardians. The C. to G., 206 Goldhawk Road, Shepherd's Bush, W.
WARD SISTER (£30), Bermondsey Guardians. E. Pitts Fenton, C. to G., 283 Tooley Street, S.E. Nov. 11.

### Situations Vacant.

ASSISTANT COOK (£20), Hammersmith Guardians. The C. to G., 206 Goldhawk Road, Shepherd's Bush, W. Nov. 13.
ASSISTANT ENGINEER (£45), Hammersmith Guardians. The C. to G., 206 Goldhawk Road, Shepherd's Bush, W. Nov. 13.
ASSISTANT LAUNDRESS (£30), Kingston Union. Mr. C. W. Dash, C. to G., Kingston-on-Thames.
ASSISTANT LAUNDRESS (£20), Greenwich Union. Mr. T. Cutter, C. to G., East Greenwich.
COOK (£25), Bosmere and Claydon Union. Mr. R. M. Cook, C. to G., 6 Providence Street, Ipswich. Nov. 13.
COOK (£25), Bosmere and Claydon Union. Mr. R. M. Cook, C. to G., 6 Providence Street, Ipswich. Nov. 13.
FEMALE COOK (£27 10s.), Preston Union. Mr. J. Clarke, C. to G., Preston. Nov. 23.
FEMALE GENERAL ASSISTANT (£20), Pocklington Union. Mr. T. Robson, C. to G., Pocklington. Nov. 17.
HEAD LAUNDRESS (£34), Newport Mental Hospital. Medical Superintendent of the Hospital, Caerleon, Mon.
LAUNDRESS (15s. weekly), Basford Union. Mr. H. Stone, C. to G., Basford, Notts.
NURSERY AND YARD ATTENDANT (£26), Basford Union. Mr. H. Stone, C. to G., Basford, Notts.
PORTER AND COOK (married couple) (£22 10s. each), Wantage Union. Mr. E. B. Ormond, C. to G., Wantage. Nov. 11.
PORTRESS AND LAUNDRY SUPERINTENDENT (£20), Runcorn Union. Mr. G. F. Ashton, C. to G., Runcorn.
RELIEF MAID (£20), Bermondsey Guardians. Matron of the Infirmary, Lower Road, Rotherhithe.
WARDMAID (£18), Great Yarmouth Union. Mr. F. Burton, C. to G., Great Yarmouth. Nov. 12.
WORKING LAUNDRESS (£20), Witney Union. Mr. T. J. Finnis, Master of the Workhouse, Witney, Oxon. Nov. 13.

### Institutional and Administrative Appointments.

OPHTHALMIC SISTER (£30), King Edward VII.'s Hospital, Cardiff. Apply Matron. Nov. 18.
MATRON (£50), Fleetwood Cottage Hospital. Hon. Secretary, Milton Lodge, Fleetwood. Nov. 12.
MATRON (£40), Llangollen Cottage Hospital. Hon. Secretary. Nov. 25.
MATRON (£45), Exmouth Cottage Hospital. Hon. Secretary. Nov. 20.
NURSE (£18), Surrey County Asylum. Medical Superintendent of the Asylum, Netherne, Merstham.
NIGHT NURSE (£25), Surrey County Asylum. Medical Superintendent of the Asylum, Netherne, Merstham.
NIGHT SISTER (£30), Hartlepools Hospital. Apply Matron. Nov. 17.
OPHTHALMIC SISTER (£30), King Edward VII.'s Hospital, Cardiff. Matron. Nov. 18.
SISTER (£30), Royal Eye and Ear Hospital, Bradford. Matron.
STAFF NURSE (£28), Colwyn Bay Cottage Hospital. Hon. Secretary, Nov. 22.

### Situations Vacant.

WARDMAIDS (£18), M.A.B. Matrons, Western Hospital, Fulham, S.W.; North-Western Hospital, Hampstead, N.W.; and the Southern Hospital, Dartford.
ASSISTANT COOK (£24), M.A.B. Matron, Queen Mary's Hospital, Carshalton.
COOK-GENERAL (£22), M.A.B. Matron, Millfield, Rustington, near Littlehampton.

### Miscellaneous Vacancies.

INSPECTOR OF MIDWIVES, Berks County Council. J. T. Morland, Clerk, The Forbury, Reading. Nov. 23.
SCHOOL NURSE (£75), Tottenham Education Committee. W. Mallinson, Clerk, Education Offices, S. Tottenham. Nov. 11.
SCHOOL NURSE (£85), Notts Education Committee. H. Handford, M.D., Shire Hall, Nottingham. Nov. 18.
SCHOOL NURSE (£65), Exeter Education Committee. H. Morgan, clerk, Education Offices, Exeter. Nov. 25.
SEWING MISTRESS FOR GIRLS' INDUSTRIAL SCHOOL (£35), Leeds Education Committee. Mr. J. Graham, Secretary for Education, Leeds.

### Municipal Vacancies.

### Appointments Vacant.

ASSISTANT NURSE (£24), Metropolitan Asylums Board. Matron, Park Hospital, Hither Green, S.E.
ASSISTANT NURSE (temporary) (£27), Watford Isolation Hospital. Matron.
ASSISTANT NURSE (£20), Stafford Isolation Hospital. The Town Clerk, Stafford.
ASSISTANT NURSES (£20), Metropolitan Asylums Board. Matrons, Western Hospital, Fulham, and Brook Hospital, Woolwich.

Assistant Nurse (£25), Duntocher Fever Hospital, near Glasgow. Apply Matron.

Caretaker and Matron (married couple) (state salary), Stanhope and Weardale Joint Hospital Committee. Mr. F. H. Thompson, Clerk to the Committee, Stanhope, Durham. Nov. 13.

Cemetery Superintendent (£90), West Bromwich T.C. Mr. A. Wickham, Town Clerk, West Bromwich. Nov. 11.

Chief Inspector of Weights and Measures (£180), Bradford T.C. Mr. F. Stevens, Town Clerk, Bradford. Nov. 15.

Clerk of Works (Sewerage) (£3 3s. weekly), Scunthorpe and Crosby Joint Sewerage Board. Mr. G. E. Davy, Clerk to the Board, High Street, Scunthorpe. Nov. 21.

Health Visitor (£75), Stoke-on-Trent Corporation. E. B. Sharpley, Town Clerk, Stoke-on-Trent. Nov. 23.

Health Visitor (£100), Finsbury B.C. Mr. G. W. Preston, Town Clerk, Rosebery Avenue, E.C. Nov. 20.

Health Visitor and School Nurse (£90), Norwich T.C. Dr. H. Cooper Pattin, Medical Officer of Health, Norwich. Nov. 11.

Inspector of Slaughter Houses and Meat (£150), Liverpool T.C. Mr. E. R. Pickmere, Town Clerk, Liverpool. Nov. 11.

Nurse (£35), Brandon and Byshottles U.D. Council. A. A. Luxmoore, 5 North Bailey, Durham. Nov. 13.

Probationer (£14), Lancaster Corporation. Apply Matron, Sanatorium, Lancaster.

Probationer (£16), Brighton Borough Sanatorium. Matron.

Probationers (£18), Metropolitan Asylums Board. Matron, Brook Hospital, Woolwich.

Staff Nurses (£26), Metropolitan Asylums Board. Matrons, Western Hospital, Fulham, and Brook Hospital, Woolwich.

Staff Nurse (£25), Leek Isolation Hospital. Clerk to the U.D. Council, Town Hall, Leek.

Surveyor (£120), Halesowen R.D.C. Mr. E. H. Grove, Clerk to the Council, Great Carnbow, Halesowen. Nov. 20.

Water Engineer and Manager (£300), Salford T.C. The Town Clerk, Salford.

### Situations Vacant.

Caretakers for Isolation Hospital, Atherstone R.D.C. Mr. W. A. Hatton, Clerk to the Council, Atherstone.

Cook (£28-30), Coventry City Hospital. Apply Matron. Dec. 6.

Housemaid (£18), Colne and Holme Fever Hospital, Meltham. Matron.

### The Scientific Press Publications

### INSTITUTIONAL WORKS.

**28 and 29 Southampton Street, Strand, London, W.C.**

Send for complete Catalogue.

### Appointments, Resignations and Vacancies.

### POOR LAW APPOINTMENTS.

Tunnicliffe, Mr. E. T., First Assistant Clerk, Dewsbury Union.
Ford, Mr. P. C. Second Assistant Clerk, Dewsbury Union.
McCloghey, Miss H., Resident Assistant Medical Officer at the Workhouse Infirmary, Toxteth Park Township.
Hodgson, Mr. R., Master of the Workhouse, Northallerton Union.
Bell, Miss L., Matron of the Workhouse, Northallerton Union.
Laird, Mr. W. H., Master of the Workhouse, Knighton Union.
Laird, Mrs. E. H., Matron of the Workhouse, Knighton Union.
Brookes, Miss E., Assistant Schoolmistress, Walsall and West Bromwich School District.
Alder, Miss A. A., Superintendent Nurse, Malling Union.

#### Charge Nurses.

Parry, Miss M. J., Bridgend and Cowbridge Union.
Brewin, Miss B. A., St. Marylebone Parish.
Burns, Miss E., St. Marylebone Parish.
Storey, Miss J. N., St. Marylebone Parish.
Cook, Miss A., St. Marylebone Parish.
Taylor, Miss L., West Bromwich Union.
Tucker, Miss L., Holborn Union.
Gallagher, Miss R., Fulham Parish.
Brooks, Miss A. G., Woolwich Union.
Croddan, Miss M. W., Lambeth Parish.
Williams, Miss A., Lambeth Parish.

#### Nurses.

Gibson, Mr. E. J., Wandsworth Union.
Harris, Miss E., Hastings Union.
Greenard, Miss E. M., Tendring Union.
Mason, Miss A. H., Fulham Parish.
Suggate, Miss M. L., Holborn Union.
Howse, Miss E. J., Freebridge Lynn Union.
Hooper, Mr. G. H., Lambeth Parish.

#### Assistant Nurses.

Cadwell, Miss F. L., Wandsworth Union.
Dix, Miss M., Halstead Union.
Procter, Mrs. N., Macclesfield Union.

#### Probationer Nurses.

Reside, Miss F. I., Carlisle Union.
Hawkins, Miss A. M., Wandsworth Union.
Whitfield, Miss A., Wandsworth Union.
Clitheroe, Miss E. G., Wandsworth Union.
O'Shea, Miss M. E., Wandsworth Union.
Lawless, Mrs. M. A. C., Warrington Union.
Martin, Miss J., Warrington Union.
Allen, Miss F. E., Hackney Union.
Rees, Miss E., Bridgend and Cowbridge Union.
Phillips, Miss H., Bridgend and Cowbridge Union.
Williams, Miss M. L., Bridgend and Cowbridge Union.
Pepper, Miss C. M. G., St. Marylebone Parish.
Bishop, Miss E., St. Marylebone Parish.
Hoffacher, Miss L., St. Marylebone Parish.
Clarke, Miss E., St. Marylebone Parish.
Pain, Miss L. L., St. Marylebone Parish.
Mort, Miss E. A., St. Marylebone Parish.
Holmes, Miss L. E., St. Marylebone Parish.
Livington, Miss G. I., St. Marylebone Parish.
Frost, Miss F., St. Marylebone Parish.
Hersee, Miss I. C., Northampton Union.
Elphick, Miss M. F., Lewisham Union.
Jones, Miss A. M., Pontypridd Union.
Mathias, Miss M., Pontypridd Union.

#### Other Officers.

Lawson, Mr. J. W., District Medical Officer, St. Pancras Parish.
Broomhead, Mr. S. A., Vaccination Officer, St. Pancras Parish.
Thomas, Mr. W. R., Relieving Officer and Vaccination Officer, Leominster Union.
Botham, Mr. J. W., Storekeeper and Distributor of Outdoor Relief in Kind, St. Pancras Parish.
Meads, Mr. P., Assistant Relieving Officer, Fulham Parish.

### POOR LAW RESIGNATIONS.

Gould, Mr. P. G., Second Assistant Clerk, Southampton Union.
Beadles, Mr. J. N., Third Resident Assistant Medical Officer at the Bromley Sick Asylum, Poplar and Stepney Sick Asylum District.
Tuffin, Mr. A. H., Superintendent of the Southbourne Children's Home, Westbourne Union.
Laird, Mr. W. H., Master of the Workhouse, Haltwhistle Union.
Laird, Mrs. W. H., Matron of the Workhouse, Haltwhistle Union.
Cross, Miss F. L., Assistant Matron at the Infirmary, Southampton Union.
Smith, Miss M. L., Industrial Trainer, Downham Union.
Cooper, Miss G., Charge Nurse, Huddersfield Union.
Tuffin, Mrs. M. A., Nurse, Westbourne Union.
Bennett, Miss K., Nurse, Newbury Union.
Maw, Miss B., Assistant Nurse, Exeter Parish.
Travis, Miss E., Assistant Nurse, Exeter Parish.

#### Probationer Nurses.

Clough, Miss N., York Union.
Ibberson, Miss A., Haslingden Union.
Davies, Miss G., Bedwellty Union.
Lane, Mr. C. H., Relieving Officer, Worcester Union.

### PUBLIC HEALTH APPOINTMENTS.

#### Inspectors of Nuisances.

Harris, Mr. F., Borough of Penryn.
Sheldon, Mr. W., Painscastle Rural District.
Wynne, Mr. W., Borough of Welshpool.
Roberts, Dr. W. H., has been appointed Analyst, Borough of Preston.
Bolsdon, Mr. R. E. C., Solicitor, Barnstaple, has been appointed Clerk to the Council, Barnstaple Rural District.

---

Dodds, Mr. I. R., Inspector of Nuisances, Doncaster Rural District, is deceased.
Thompson, Mr. G. S., Inspector of Nuisances, Sedgefield Rural District, has resigned.

